# Alien plant invasions of protected areas in Java, Indonesia

**DOI:** 10.1038/s41598-017-09768-z

**Published:** 2017-08-24

**Authors:** Michael Padmanaba, Kyle W. Tomlinson, Alice C. Hughes, Richard T. Corlett

**Affiliations:** 1 Center for Integrative Conservation, Xishuangbanna Tropical Botanical Garden, Chinese Academy of Sciences, Menglun, Mengla Yunnan 666303 China; 2 University of Chinese Academy of Sciences, Beijing, 100049, China

## Abstract

Alien plants are invading protected areas worldwide, but there is little information from tropical Asia. Java has the longest record of human occupation in Asia and today supports 145 m people. Remnants of natural ecosystems survive in 12 small National Parks surrounded by dense human populations, making them highly vulnerable to invasions. We surveyed eight of these, along a rainfall gradient from lowland rainforest with >3000 mm annual rainfall to savanna with <1500 mm, and a 0–3158 m altitudinal gradient, using 403 10 × 5 m plots along trails. We found 67 invasive alien plant species, of which 33 occurred in only one park and two (*Chromolaena odorata* and *Lantana camara*) in all. Historical factors relating to plant introduction appeared to be as important as environmental factors in determining which species occurred in which park, while within parks canopy cover and altitude were generally most influential. Spread away from trails was only evident in open habitats, including natural savannas in Baluran National Park, threatened by invasion of *Acacia nilotica*. Existing control attempts for invasive aliens are reactive, localized, and intermittent, and insufficient resources are currently available for the early detection, prompt action, and continued monitoring that are needed.

## Introduction

Alien plant invasions of protected areas are increasing worldwide and are widely recognized by managers as a major threat^1^. The literature on this problem, however, has a strong geographical bias, with most studies done in North America, Europe, South Africa, and Australia, and far fewer in the tropics. Moreover, most studies in the tropics have either been carried out on oceanic islands, where even undisturbed forests can be transformed by alien plant invasions^2, 3^, or in continental savannas^1, 4, 5^ and wetlands^6, 7^. In contrast, current evidence suggests that continental tropical forests may be relatively resistance to plant invasions, unless disturbance opens up the canopy^8–10^. Tropical protected areas are typically under-resourced and managers must focus on the major current threats. This study therefore assessed the patterns of alien plant invasion into protected areas on the continental island of Java in order to assess the magnitude of the threat.

Java is the fifth largest island in the sprawling Indonesian archipelago, but it is surrounded by shallow seas and for more than half of the Pleistocene it was a southeastern extension of mainland Asia. This accessibility, coupled with its varied natural environments, probably accounts for Java having the longest record of human occupation in Asia. *Homo erectus* arrived more than 1.5 million years ago, followed by modern *H. sapiens*, probably by at least 40,000 years ago^11^. Farming started at least 3000 years ago^12^ and over the last 1500 years the fertile volcanic soils have supported a succession of Hindu-Buddhist kingdoms, Islamic sultanates, and, from the 17^th^ century, a Dutch colony^13^. The resulting increase in human populations was responsible for the extinction of sun bears, tapirs, elephants, and most recently tigers (in the 1970s) from Java^14^. Today, 71 years after Indonesian independence, Java’s 127,000 km^2^ supports more than 145 million people^15^, making it the most populous island in the world. Rapid economic development in Indonesia in the last decade has been concentrated in Java, which has seen increased prosperity, expanding urbanization, and massive infrastructure construction. Java has thus been subject to longer and more intensive human influence than any other area in equatorial Southeast Asia^11, 13, 14^.

The long period of human settlement, extremely high population density (>1000 people km^−2^), and rapid economic growth has transformed the Javan landscape, with most cultivable land cleared of its natural vegetation cover by the middle of the 20^th^ century^14, 16^, and an increasing area since then covered in human settlements, industrial developments, and infrastructure. However, Java also has 12 well-protected National Parks that contain the last significant remnants of the major natural ecosystem types on the island. Although these are all small^17^, and were all subject to human and volcanic disturbances in the past, they still support populations of Java’s remaining large vertebrates (primates, deer, banteng) whose visibility attests to the low hunting pressure, as does the survival of the last Javan rhinoceros population on Earth in Ujung Kulon National Park. The more accessible parks are very popular at weekends with Javanese from the cities, and visitor numbers are increasing rapidly (Table 1), but human recreational pressures appear to be generally well-managed. All these parks, however, exist as widely separated islands in a highly-modified, densely populated, agricultural matrix, with little or no buffer between the two.

**Table 1:** Numbers of visitors 2011–2015 to the eight National Parks in Java surveyed in this study. Data from Directorate of Forest Conservation and Environmental Services at the Ministry of Environment and Forestry.

National Park	2011	2012	2013	2014	2015
Ujung Kulon	6,691	7,433	9,248	12,429	15,150
Gunung Gede Pangrango	88,953	85,486	139,767	165,823	154,948
Gunung Merapi	193,779	72,797	100,965	200,308	254,000
Gunung Merbabu	26,789	25,662	25,012	11,220	42,828
Bromo Tengger Semeru	125,471	275,874	545,648	571,158	474,011
Meru Betiri	2,443	3,342	9,055	60,092	89,071
Alas Purwo	90,875	100,315	121,818	133,557	132,220
Baluran	28,851	32,674	39,874	60,385	93,054

The small size of the parks, their isolation, and the heavy human pressures are all expected to increase the risk of biological invasions. A recent global review found that invasive alien plants are present in almost all protected areas, although they are generally less prominent than in unprotected areas^1^. As a case study, the Javan parks have the advantage that they are spread across the full range of physical environments and natural ecosystems in Java, from lowland and montane rainforests in the west to dry savannas in the east. Moreover, these are also fairly representative of similar ecosystems across Southeast Asia^11^. Java is extreme in the magnitude of the human pressures, but protected areas across the tropics are becoming increasingly isolated as human populations on their borders increase^18^. Previous studies in Java have looked at individual parks^19–23^, but no previous study has made use of the full range of terrestrial environments represented in the park system. This study therefore attempts to answer three main questions about invasive alien plant species in Java’s National Parks. 1. Which alien plant species are invading and where? 2. What factors determine which species invade which parks? 3. What factors control their distributions and abundances within parks?

## Results

Most environmental variables were associated with the west-east gradient of declining rainfall and/or the 0–3158 m gradient in altitude (Supplementary Table S1). Ujung Kulon is the wettest park, with a mean annual rainfall of 3515 mm, while Baluran is the driest, with only 1095 mm. Alas Purwo is the warmest, with a mean temperature of 27.8 °C and Gunung Merbabu the coolest, with a mean of 11.6 °C. At the plot scale, mean temperature ranged from 8.9 °C in the coolest plot in shrubland at 3152 m on Gunung Merbabu to 30.5 °C in the warmest plot in savanna at 68 m in Baluran. In Gunung Gede Pangrango, mean temperature declined regularly with altitude while minimum and maximum temperatures declined significantly, but less regularly (Supplementary Fig. S1). No data logger recorded frost during the study period. Frosts have been reported to occur in Java above 1500 m, but only in depressions and never on slopes^24^. Humidity declined but not significantly. Most soil variables varied widely within parks and the ranges overlapped among parks (Supplementary Table S1). Soils were generally sandy and acidic, except in the two driest parks, where the soils were generally alkaline. In Gunung Gede Pangrango, soil organic carbon, silt, and nitrogen increased significantly with altitude, while clay declined, but the scatter was large (Supplementary Fig. S2). All parks had sample plots with low and high tree cover, but the mean cover was highest in Ujung Kulon and lowest in Baluran.

A total of 67 invasive alien plant species (IAS; Supplementary Table S2) were found in the sample plots, with 261 (64.8%) of the 403 plots containing at least one. Seven species were found in only a single plot and almost half (33) only in one park. The families Asteraceae (18 species), Fabaceae (10), Solanaceae (7), and Euphorbiaceae (5) accounted for most species. There were four species of trees, all in the Fabaceae, and four lianas, while the rest were herbs (39) or shrubs (20). A large majority of species were from the Neotropics (53), followed by the temperate zone (7; all from the northern hemisphere except for *Fuchsia magellanica* from temperate South America), Africa (4), India (1), and Australia (1) (Supplementary Table S3). The temperate zone species were all found only or largely above 2000 m. More than half the total species (40) were already reported in Java in the nineteenth century (Supplementary Table S3). It is noticeable, however, that *Ageratina riparia* (first recorded in Java in 1963) and *Chromolaena odorata* (1940s) are among the most abundant IAS in the parks they have reached.

The numbers of species recorded in each park ranged from eight in Ujung Kulon and Alas Purwo, which are on isolated peninsulas at opposite ends of Java, to 27 in Meru Betiri (Table 2), and was not correlated with the number of plots sampled (Spearman’s ρ = −0.11, p = 0.80). Only two species, *Chromolaena odorata* and *Lantana camara*, were found in all eight parks (Supplementary Table S2), and in Gunung Gede Pangrango they were both only found outside the plots at low elevation. *C. odorata* was also among the three most frequent species in five parks (Table 2). *Clidemia hirta* was found in all but the two driest parks, but was sparsely distributed everywhere. *Elephantopus scaber*, *Hyptis capitata*, and *Passiflora foetida* were in all lowland parks but none of the mountain parks, while *Ageratina riparia* and *Austroeupatorium inulaefolium* were in all mountain parks but none of the lowland parks. Moreover, *P. foetida* was the most frequently recorded species in lowland Baluran, while *A. riparia* was the most frequent in all the mountain parks (Table 2). *Acacia decurrens* was frequent in three mountain parks but absent from all the others. Some of the species found in only one park—*Acacia nilotica* in Baluran, and *Brugmansia candida* and *Passiflora ligularis* in Gunung Gede Pangrango—were frequent where they occurred. Indeed, *A. nilotica* now covers more than 60 km^2^ of Baluran after being first planted there in 1969^22^.

**Table 2:** Invasive alien plant species number and abundance in the plots (each 10 m × 5 m) surveyed in eight National Parks in Java. Alien trees and lianas in the plots were counted and alien shrub and herb covers were estimated.

National Park	Land area (km^2^)	Total plots	Total alien spp.	Plots with aliens	Tree/ liana count	Shrub/ herb cover	Most common aliens	N of Plots
Ujung Kulon	786.2	55	8	6	0.02	0.41	*Hyptis capitata*	2
							*Lantana camara*	2
Gunung Gede Pangrango	219.8	32	14	24	0.19	15.14	*Ageratina riparia*	15
							*Brugmansia candida*	5
							*Solanum americanum*	4
							*Passiflora ligularis*	4
Gunung Merapi	64.1	48	13	38	4.31	18.69	*Ageratina riparia*	29
							*Chromolaena odorata*	21
							*Acacia decurrens*	15
Gunung Merbabu	57.3	38	14	31	1.21	21.49	*Ageratina riparia*	30
							*Austroeupatorium inulaefolium*	21
							*Acacia decurrens*	15
Bromo Tengger Semeru	502.8	94	23	72	1.34	28.10	*Ageratina riparia*	46
							*Chromolaena odorata*	43
							*Acacia decurrens*	21
Meru Betiri	554. 0	14	27	14	4.36	12.68	*Eleutheranthera ruderalis*	12
							*Mikania micrantha*	11
							*Chromolaena odorata*	9
Alas Purwo	430.2	41	8	13	0.07	2.28	*Chromolaena odorata*	13
							*Hyptis capitata*	6
Baluran	239.4	81	24	63	3.33	5.4	*Passiflora foetida*	24
							*Acacia nilotica*	21
							*Chromolaena odorata*	18

No invasive species were found on eight 50-m transects from trails into the forest in Ujung Kulon, and few penetrated more than 10 m from the trails in Gunung Gede Pangrango. In Alas Purwo and Baluran, invasive species were rare or absent on transects with high canopy cover, but in areas of with low or no canopy cover all the common invasives spread to the limits of the transects. In Baluran, *Acacia nilotica*, *Ageratum conyzoides*, *Hyptis suaveolens*, *Mimosa pudica*, and *Passiflora foetida* were particularly common on the transects.

**Figure 1:**
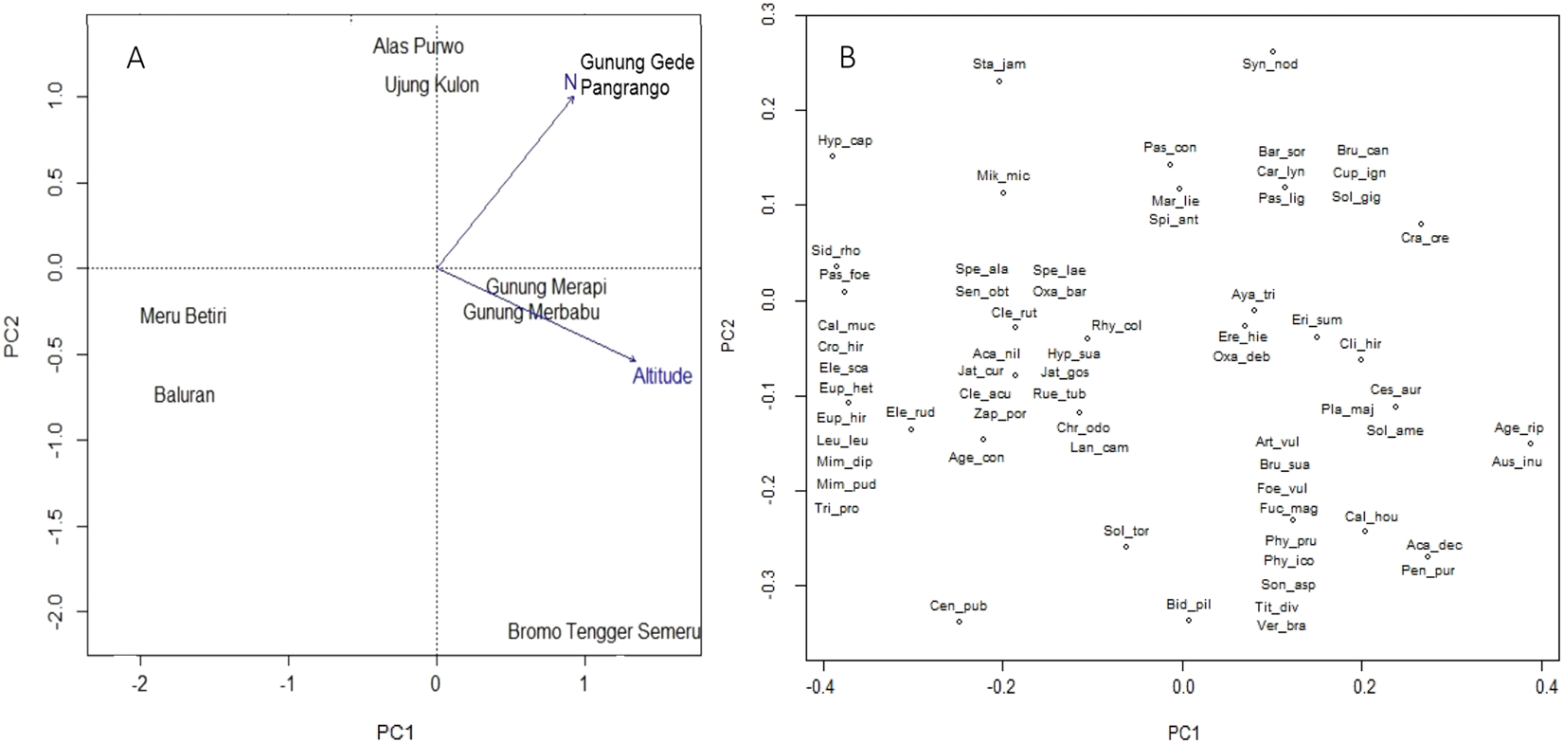
PCA ordination of the park by alien species matrix showing (**A**) the eight parks and (**B**) the alien species. The vectors show the predictors (altitude and soil total N) significantly related to the ordination space at p < 0.05. Both plots show the same ordination space, but the vectors are omitted and the scale expanded in the species ordination for clarity. Species acronyms are the first three characters of the genus and specific epithet.

**Figure 2:**
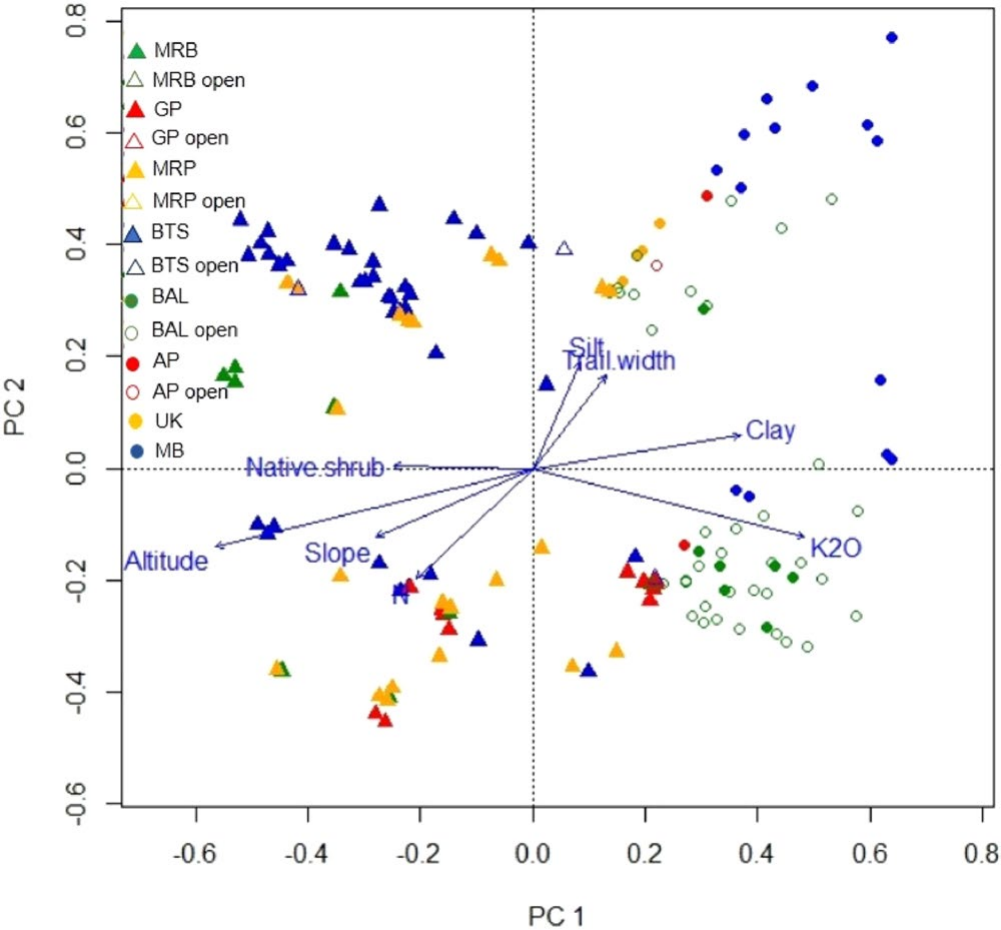
PCA ordination of the plot by alien species matrix (excluding plots with no alien species). Plots in mountain parks are represented by triangles, from the lowland parks by circles, from closed forest by filled symbols, and from open habitats by unfilled symbols. The vectors show the predictors (altitude, slope, total N, potassium, clay, silt, trail width, and native shrub cover) significantly related to the ordination space at p < 0.05. UK = Ujung Kulon; GP = Gunung Gede Pangrango; MRP = Gunung Merapi; MRB = Gunung Merbabu; BTS = Bromo Tengger Semeru; MB = Meru Betiri; AP = Alas Purwo; BAL = Baluran.

In agreement with the patterns described above, the PCA ordination of the park × species matrix separated the four lowland parks from the four mountains, and Gunung Gede Pangrango from the other mountains (Fig. 1A). The first two principal components accounted for more than half of the variation in the data, where PCA1 explained substantially more variation (37.8%) than PCA2 (17.3%). Only altitude and soil total nitrogen were significantly correlated with these two axes. The species ordination separated three clusters, representing lowland-only species (all of tropical origin), high altitude species (tropical or temperate), and species only found on Gunung Gede Pangrango (tropical or temperate), with the more widespread species spread between them (Fig. 1B). The PCA on the plot × species matrix, excluding plots without IAS, showed a similar pattern to the PCA for park × species, except that there was considerable overlap, with some plots from different parks sharing similar invasive species compositions, and the first two principal components accounted for only 15.8% and 11.8% of the variance (Fig. 2). Significantly associated environmental variables were altitude, slope, soil total N, available potassium, clay content, silt content, trail width, and native shrub cover.

Not surprisingly, given the large differences between parks in the range of values shown by each environmental variable, the drivers identified by the park-level generalized linear mixed models (GLMM) varied among parks (Table 3). However, canopy cover had a significant negative impact on the numbers and abundances of IAS in most lowland parks, while altitude had a significant negative impact in most mountain parks, as well as Baluran and Alas Purwo. Other patterns were more idiosyncratic, including positive effects of trail width on number of alien species in Baluran and total nitrogen in Bromo Tengger Semeru, and negative effects of slope and total nitrogen in Alas Purwo, and clay in Ujung Kulon and Gunung Merbabu.

**Table 3:** Environmental predictors (with estimated coefficient) of the total number of alien species (N) (Table 2), numbers of individual alien trees (Tr) and lianas (Li), and covers of alien shrubs (Sh) and herbs (He) in surveyed plots in eight National Parks in Java. Bold numbers are significant regression coefficients (p < 0.05). abs = absent; b-bin = betabinomial.

Predictor	Ujung Kulon					Meru Betiri				
	N	Tr	Li	Sh	He	N	Tr	Li	Sh	He
Intercept	**−2.37**	abs	abs	**−7.20**	**−9.20**	**1.68**	abs	**1.13**	**−4.27**	**−2.64**
Altitude	0.73				**1.21**	**−0.30**		**−0.78**	**1.47**	
Canopy cover	**−0.49**			**−1.02**	**−1.37**				0.83	**−0.56**
Shrub cover								**1.07**		
Native shrub cover				**−**0.87	**−1.49**				**−0.88**	**−0.47**
Trail width					**−**0.95			**−0.90**	**8.26**	0.43
Clay	**−**0.97							**1.42**	**−15.82**	**−**0.34
Total N								**−1.31**	**18.22**	
Available K									**13.68**	
error type	Poisson			b-bin	b-bin	Poisson		Poisson	b-bin	b-bin
**Predictor**	**Alas Purwo**					**Baluran**				
	**N**	**Tr**	**Li**	**Sh**	**He**	**N**	**Tr**	**Li**	**Sh**	**He**
Intercept	**−1.23**	abs	abs	**−5.10**	**−6.53**	**−**0.21	**−**0.67	0.49	**−5.23**	**−7.08**
Altitude						**−**0.31	**−2.18**	**−0.80**		
Slope	**−0.78**			**−1.18**	**−**1.32	**−**0.13		**0.68**	**−**0.28	
Canopy cover	**−**0.39			**−0.89**		**−0.29**	**−0.69**		**−0.55**	
Shrub cover							**−1.44**	**0.70**		
Native shrub cover										**−**0.33
Trail width						**0.61**		0.22	**0.97**	**1.80**
Total N	**−**0.51			**−**0.65	**−**2.38	**−0.24**			**−**0.27	
Available K				0.53						
error type	Poisson			b-bin	b-bin	Poisson	nbinom	nbinom1	b-bin	b-bin
**Predictor**	**Gunung Gede Pangrango**					**Gunung Merapi**				
	**N**	**Tr**	**Li**	**Sh**	**He**	**N**	**Tr**	**Li**	**Sh**	**He**
Intercept	**−**18.85	abs	abs	68.75	**−**49.70	**0.49**	**−**0.50	abs	**−2.23**	**−2.89**
Altitude	**−0.96**			**−**0.74	**−**0.48	**−0.62**	**−1.84**		**−1.04**	**−0.82**
Slope				**−**4.98						
Canopy cover				**5.84**			**1.70**			
Shrub cover						**0.36**				
Native shrub cover				**−1.63**		**−0.50**			**−0.79**	**−0.39**
Trail width							**−2.71**			
Total N							**2.18**			
Available P	0.39			**−3.17**	**0.98**					
Available K				**13.38**						
error type	Poisson			b-bin	b-bin	Poisson	Poisson		b-bin	b-bin
**Predictor**	**Gunung Merbabu**					**Bromo Tengger Semeru**				
	**N**	**Tr**	**Li**	**Sh**	**He**	**N**	**Tr**	**Li**	**Sh**	**He**
Intercept	0.25	**−11.64**	abs	**−11.26**	**−2.19**	**7.84**	**41.63**	abs	**23.95**	2.63
Altitude	**−0.65**	**−3.37**		**−2.73**	**−0.62**	0.0003				
Slope						0.09	**−**0.18		**0.36**	
Canopy cover						**−**0.004			**−0.01**	
Shrub cover						**0.02**	**0.03**			
Native shrub cover					**−0.44**	**−0.01**	**−0.03**		**−0.01**	**−**0.01
Trail width				**−**0.33			**−0.66**		**−**0.56	
Sand							**0.18**		**−0.22**	**−0.17**
Clay	**−0.23**									
Total N					**−**0.37	**4.26**	**29.66**			
Available K		**2.23**		**1.71**			1.24		**−1.99**	**2.09**
error type	Poisson	nbinom		b-bin	b-bin	Poisson	nbinom1		b-bin	b-bin

## Discussion

Invasion biologists have recognized the existence of multiple barriers to invasion that must be overcome before a species becomes truly invasive^25^. The first of these is geographical: an invasive species must be transported, deliberately or accidentally beyond its native range. None of the species identified as alien in this study are native to Java so all must have crossed marine barriers to get there. For species that can establish and spread in the human-dominated matrix, reaching Java is enough to provide an opportunity for invasion. Many of the species recorded in this study, however, appear to be rare or absent in the matrix (personal observations) and thus require separate accidental or deliberate introduction to each park before they can become invasive. Consistent with this, almost half the alien species were recorded in only one park, despite the considerable overlap among parks in environmental conditions (Supplementary Table S1). All these species have self-sustaining wild populations (i.e. they are naturalized) and, in at least some cases, they have spread widely in the parks where they occur (i.e. they are invasive). This suggests that historical factors—in particular, whether or not the species was introduced there—are at least as important as environmental factors in determining which species become invasive in each park.

In support of this role for historical factors, the sources of park-specific invasions are known for seven species. These include the deliberate planting of *Acacia nilotica* as a fire break in Baluran in 1969^22^, and the spread of *Acacia decurrens* from state-owned forest concessions in Gunung Merapi, Gunung Merbabu, and Bromo Tengger Semeru (personal communications). Five species apparently escaped into Gunung Gede Pangrango from the adjacent Cibodas Botanical Garden, including *Bartlettina sordida*, *Cestrum aurantiacum*, two *Brugmansia* spp., and *Passiflora ligularis* ^20, 26, 27^. All but one of these species are still confined to the park or parks into which they were historically introduced and where they are common, despite the occurrence of similar environments in other parks (Supplementary Table S1). *C. aurantiacum* also occurs in Bromo Tengger Semeru, presumably from a separate introduction in view of the wide separation between this and Gunung Gede Pangrango.

In contrast, the two species found in all parks, *Chromolaena odorata* and *Lantana camara*, are both very widespread in the matrix between parks (personal observations; http://gbif.org/occurrence) and both tolerant of a wide range of frost-free climates. The three species only in all lowland parks, *Elephantopus scaber*, *Hyptis capitata*, and *Passiflora foetida*, and their mountain park counterparts, *Ageratina riparia* and *Austroeupatorium inulaefolium*, are also present in the matrix (personal observations), but their distributions suggest strong thermal control of their invasiveness.

In contrast, once a species has become established in a park, we expected environmental factors to control its establishment and spread. Time since first introduction may also limit the distributions of some species, but many have been in Java a long time—all were first recorded by 1963—and most are dispersed by wind (including most Asteraceae), small birds (e.g. *Clidemia hirta*, *Lantana camara*, *Passiflora* spp., *Solanum* spp.) and/or mammals (e.g. large herbivores for *Acacia nilotica* ^21^ and, presumably, arboreal mammals for *Passiflora ligularis*). Human visitors probably also transport some seeds on clothing^28^ and others possibly in mud on shoes^29^. This has not been investigated in Java, but a recent review included six species from our study (*Ageratum conyzoides*, *Artemisia vulgaris*, *Bidens pilosa*, *Mimosa pudica*, *Sida rhombifolia*, and *Sonchus asper*) on its list of species recorded on clothing^28^.

The relationships with environmental variables identified by the GLMMs are varied and complex, but canopy cover stands out as a negative influence on invasions in most lowland parks and altitude as a negative influence in two lowland and most mountain parks. The influence of soil variables was also sometimes strong, but these variables were interpolated over complex topography from soil analyses in only 35 of 403 plots, so these relationships need further study. The strong influence of canopy cover in the lowlands is not surprising, since *Clidemia hirta* is the only common shade-tolerant, lowland, invasive species. The park with the densest canopy cover, Ujung Kulon, also had the fewest invasive species and the smallest proportion of invaded plots. The mountain parks, in contrast, are generally more forested and their invasive floras include additional, relatively shade tolerant species, including the widespread *Ageratina riparia*. The strong influence of altitude in the mountains is also unsurprising since, except for some early Dutch experiments with temperate plants on Gunung Gede Pangrango^26^, alien invasive species must establish first at low altitudes and then spread upwards^30^. A consequence of this is that, as on mountains elsewhere^30, 31^, the most widespread invasive species at high altitudes in Java are not high-elevation specialists, but two species with a very broad range of environmental tolerance, *Ageratina riparia* and *Austroeupatorium inulaefolium*.

Invasive species along trails may be an aesthetic problem, particularly if visitors are aware of their status, but they are of major conservation concern only if they can invade the much larger areas between trails. In this case, canopy cover seems to be the critical variable. In Ujung Kulon and most parts of Alas Purwo and Baluran with high canopy cover, no invasives were found away from trails, and in well-forested Gunung Gede Pangrango few penetrated further than 10 m. In areas of Alas Purwo and Baluran with low canopy cover, in contrast, all the common invasives spread to the limits of the transects. In must be stressed, however, that the off-trail penetration of invasive alien plants needs more extensive study, including investigation of natural open areas in tree fall gaps and landslides remote from trails. It is also not clear if the current lack of invasion into closed-canopy forests results from a community-level resistance by species-rich ecosystems or is simply a reflection of the small number of shade-tolerant aliens introduced to Java.

In general, the results of this study agree with previous studies in some of the same parks^19–22^. Major exceptions can be explained by localized invasions, including *Maesopsis eminii*, which was reported common along a trail (from Bodogol) in Gunung Gede Pangrango that was not surveyed in this study^20^, and several species recorded in a savanna area at Alas Purwo^19^. This highlights a major problem with assessing plant invasions in protected areas, where broad-scale surveys like this one are an efficient way of detecting widespread species, but may miss habitat specialists or species in the early stages of invasion, both of which may require management attention. On the other hand, despite their long borders with plantations and agricultural areas, access to the Javan parks we surveyed is largely along a small number of trails, so comprehensive surveys and regular subsequent monitoring should not be an impossible task.

Invasive alien species are abundant and diverse along trails in National Parks in Java, confirming our expectation that their small size, isolation, and heavy human pressures would make them unusually vulnerable to invasion. Park staff we talked with were aware of invasive species as an actual or potential conservation issue, but informal interviews and the literature suggest a wide variation in both the degree of concern and the actions taken, if any. At Baluran, *Acacia nilotica* is an obvious conservation issue, since it threatens the open savanna habitats on which its charismatic large mammal fauna, including banteng (*Bos javanicus*) and dhole (*Cuon alpinus*), depend^22, 32, 33^. On-going control efforts use cutting followed by herbicide application to the stumps^33^. *Acacia decurrens* is the most visible problem in Gunung Merapi—where it benefitted from the massive 2010 eruption—adjacent Gunung Merbabu, and parts of Bromo Tengger Semeru, but attempts at control have not yet started. In Gunung Gede Pangrango, the liana *Passiflora ligularis* is the main focus of attention because it is believed to damage the trees it grows over, and has been controlled fairly effectively by cutting. In other parks, active control measures have been localized, such as in the savanna at Alas Purwo, or absent. In Ujung Kulon, where alien species are rare or localized, the focus has been on the native palm, *Arenga obtusifolia*, the dominance of which reduces the food plants available for the Critically Endangered Javan rhino^34^.

The limited resources available for invasive species control in National Parks in Java mean that management actions must focus on a small number of priority areas and species: conservation triage^35^. In practice, this has so far meant that most resources are used on the most abundant and conspicuous species, which are also the most difficult to control since extirpation is no longer an option. Early detection of new invasions, prompt control action where the ecology and history of a species elsewhere suggest a potential threat, and continued monitoring thereafter would be a preferable strategy^36^, but the resources for this are not currently available in most parks. However, a recent risk analysis of aliens invading Gunung Gede Pangrango identified a bamboo, *Chimonobambusa quadrangularis*, as a currently localized but potential problematic species that should be eradicated^37^. Cutting and herbicide application appear to be practical means of limiting damage from some established woody species, including *Acacia nilotica* and *Passiflora ligularis*, but experience elsewhere in protected areas suggests that biological control should be explored as a potential long-term solution^35, 36^. *Lantana camara* and *Chromolaena odorata* ^38^ have both been the subject of biological control attempts in Java, but there has been no study of the effectiveness of this in reducing invasiveness in protected areas. *Ageratina riparia* has been the subject of successful biological control campaigns in Hawaii and New Zealand^39^ and this experience could be used in Java. Possible biological control agents for *Acacia nilotica* are currently under investigation in Australia^40^.

Our expectation that National Parks in Java would be highly vulnerable to alien plant invasions is supported by the diversity and abundance of alien plant species in most of the National Parks we investigated. At present these are of major conservation concern only in naturally open habitats, such as the savanna in Baluran. However, invasive species control in Javan parks requires adequate resources and there is a risk that existing invasive problems may get worse and new ones arise undetected. Java’s National Park system is a national and global treasure and deserves maximum support: targeted funding for invasive species monitoring and management should be part of this.

## Methods

### Surveyed National Parks

**Figure 3:**
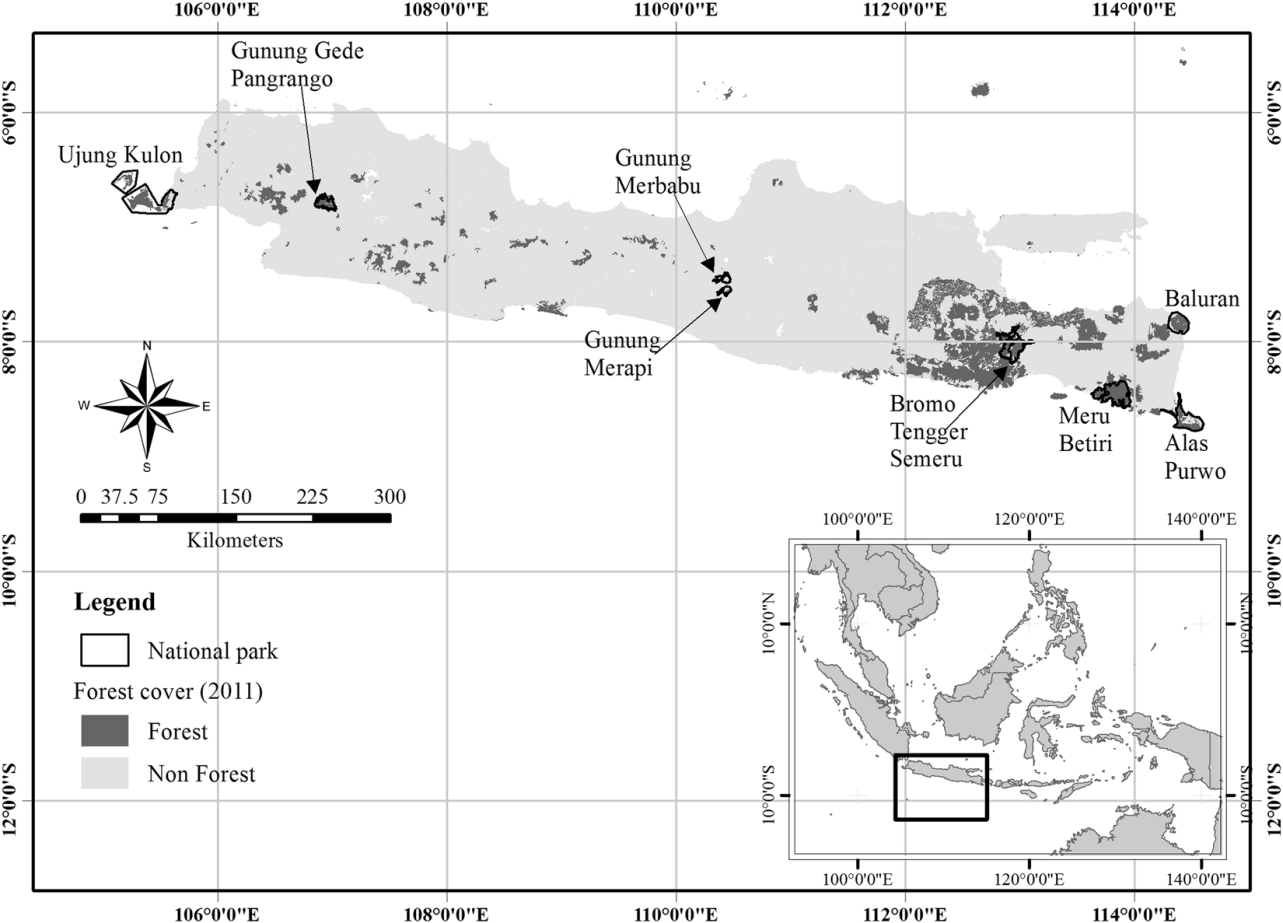
Map showing the eight National Parks sampled across Java Island, Indonesia. This map is developed using ArcGIS 10.3 (http://desktop.arcgis.com/en/). Forest cover layer is generated from Land Cover map published by Greenpeace (http://www.greenpeace.org/seasia/id/Global/seasia/Indonesia/Code/Forest-Map/en/data.html). There is no guarantee of the accuracy of this map, therefore, the use for financial or other important decisions are not advisable. Conservation area map was developed and shared under Creative Common CC BY 4.0 (https://creativecommons.org/licenses/by/4.0/legalcode) by the World Resources Institute through Global Forest Watch (www.globalforestwatch.org) and available at http://data.globalforestwatch.org/datasets/573ce69e9c0f486dbbad021317de7bab_14. Credits should be given to Directorate General of Spatial Planning, Ministry of Forestry from where the source of data (http://appgis.dephut.go.id/appgis/kml.aspx) were downloaded in 2010, processed, and provided by Greenpeace. Among all conservation area in Indonesia presented in the conservation area map, we used only those covering eight National Parks in Java where this study was undertaken.

Between September 2014 and February 2016, we surveyed eight National Parks. Ujung Kulon, Meru Betiri, Alas Purwo, and Baluran are lowland parks while Gunung Gede Pangrango, Gunung Merapi, Gunung Merbabu, and Bromo Tengger Semeru include one or more volcanoes (Fig. 3). These represent the major terrestrial ecosystems on Java: lowlands, from wet rainforest in the west to dry forests and savanna in the east, and volcanic mountains. Western Java, including Ujung Kulon, receives >3000 mm of rainfall, which declines to <1500 mm in the east, including Baluran and Alas Purwo. Dry season length and intensity also increases from west to east. Bromo, Merapi, and Semeru erupted in 2016, 2010, and 2008, while last eruptions of other volcanoes were >60 y ago^41^.

The first National Parks were designated in 1980, but all eight parks received at least partial protection decades earlier as nature reserves, wildlife sanctuaries, or protected forests. Gunung Gede Pangrango was part of the Cibodas Botanic Garden, established in 1866, which also had small experimental areas up to the summit^26^. All parks, except Ujung Kulon, are partly bordered by plantations. Visitor numbers vary widely (Table 1), but are not a good measure of human pressures because visitors are often concentrated at accessible attractions.

### Survey design

In each park, 10 × 5 m plots—403 in total—were established along roads, patrol paths, and tourist tracks. Plots started 1 m from the trail edge (or the gravel margin of roads). We started the survey at the park border or the start of natural conditions, and sampled all accessible habitat types. Plot spacing and number were determined by park area, lengths of accessible trails, and steepness of topography and environmental gradients. Gunung Merapi and Gunung Merbabu are small and steep, so plots were placed 250 m apart on alternating sides of the trails. Gunung Gede Pangrango and Bromo Tengger Semeru are large and steep, so plots were placed every 500 m, while plots were 1000 m apart in the lowlands. When several trails existed, those sampling the widest area and connecting to the highest point were chosen (Supplementary Table S4). Plot number per park ranged from 14 in Meru Betiri, where the accessible trails are relatively short, to 94 in large and complex Bromo Tengger Semeru (Table 2). Most were in forest, but open habitats were sampled in coastal areas, above the treeline on volcanoes, and in the extensive savanna in Baluran.

Invasive alien plants in each plot were recorded and grouped into trees, lianas, shrubs, and herbs. A botanist from the nearest botanical garden (Cibodas in the west and Purwodadi in the east) helped identification in the field. Species not certainly identifiable in the field were photographed, collected, and checked in herbaria. Species outside plots were recorded on an *ad hoc* basis, but we did not attempt to compile a comprehensive alien flora. In each plot, we counted the numbers of individual alien trees and lianas and estimated percent cover (to the nearest 10%) of alien shrubs and herbs. Plant names and families follow the Plant List Version 1.1 (www.theplantlist.org). The earliest record of occurrence in Java was found from historical sources^26, 42, 43^. Region of origin was checked in databases, floras, and other literature.

To assess penetration of invasive alien plants away from trails, we surveyed 70 2-m wide transects perpendicular to the trails, in Alas Purwo, Baluran, Ujung Kulon, Bromo Tengger Semeru, and Gunung Gede Pangrango. Transects were established every 1000 m in the mountains and every 2000 m in the lowlands. Where possible these were 50 m long, but some were truncated by impassable topography. Alien plant presence and abundance was recorded as in the plots, but the data is only used qualitatively in this paper.

### Environmental factors

In each plot, coordinates and elevation were measured at the center with a GPS (Garmin eTrex 30). Canopy cover was measured in the same place, with a spherical densiometer or HabitApp (an Android application on a tablet), facing in the four cardinal directions and averaged. Both methods gave similar results. Shrub cover was estimated visually. Slope was measured at plot edges using a clinometer (Suunto) and averaged. Trail width was measured with a tape.

Data loggers (iButton Hygrochron DS1923) were used to record temperature and humidity every four hours for a year in one mountain park (Gunung Gede Pangrango), one wet lowland park (Ujung Kulon), and one dry lowland park (Baluran). Two were placed at 200 m vertical intervals in Gunung Gede Pangrango and in a range of different habitats in the lowland parks. One was put in a relatively open area adjacent to the trail and the other in a shaded area. Data loggers were attached to a tree on the side that remained shaded at approximately breast height in a tea strainer to avoid contact with the bark. We had data loggers in 3 parks, but altitude for all plots, so we tested whether altitude could be used as a proxy for temperature by regressing mean monthly temperature against altitude for plots with a data logger, including Month and Park as random effects. Temperature was highly significantly correlated with altitude, giving a lapse rate of −0.004 °C per meter increase in elevation (p < 0.001, R^2^ > 0.8). We therefore used altitude in all subsequent analyses. The average annual rainfall for each park was obtained from WorldClim 1.4 (www.worldclim.org) and was consistent with available 5-year means from local climatic stations.

Soil samples were collected at 200 m vertical intervals in all parks except Gunung Merapi, for which data from adjacent Gunung Merbabu was used in analyses. Soil samples at 10 cm depth were taken from a 20 × 20 cm pit, after removing litter. In total, 35 samples were collected, oven-dried at 105 °C for 24 hours, and analyzed in the Indonesian Soil Research Institute (ISRI), Ministry of Agriculture, Bogor, following ISRI technical guidelines^44, 45^. Samples were ground with mortar and pestle, and sieved through a 2 mm mesh followed by a 0.5 mm mesh. Soil measurements included: texture (pipette method); pH (KCl and H_2_O methods)^44, 45^; organic carbon and total nitrogen^45, 46^; available phosphorus, using the method of Bray^45, 47^ for pH < 5.5 and Olsen^45, 48^ for pH > 5.6; and potassium (Morgan-Wolf extract method)^45^. Most soil variables were correlated with altitude, but with significant scatter, so measurements were interpolated to plot level using ordinary kriging in ArcGIS.

### Statistical analyses

We conducted all analyses in R statistical software^49^. To visualize how alien species were distributed across National Parks, we ran a principal components analysis (PCA) on the park × species matrix using the *rda* function in the *vegan* package^50^. We tested whether differences among parks in alien species composition were associated with differences in mean environmental conditions in each park, using the *envfit* function. Environmental predictors included were altitude, rainfall, canopy cover, native shrub cover, silt and clay fraction, and soil total nitrogen. All other parameters were highly correlated with one of these predictors. Only predictors that were significantly related to the ordination space at P < 0.05 were plotted on the ordination. We then ran a PCA on the larger plot × species matrix (excluding 142 plots with no aliens) to show variation within and between parks. We used the same predictors as in park × species matrix, but dropped rainfall, because it cannot not be robustly downscaled to plot level in rugged topography.

Differences among parks were expected to be caused, in part, by which alien species had arrived in the past, but we expected environmental controls to dominate within parks. For each park, we therefore chose between highly correlated environmental predictors (ρ > 0.7) to yield a final set included in the regression models for that park. The set of predictors included in these analyses were: altitude, canopy cover, shrub cover (used for alien trees and liana responses), native shrub cover (only used for shrub and herb responses), slope, sand and clay content, total nitrogen, available phosphorus and potassium, and trail width. We regressed the numbers of alien species, the numbers of alien tree and liana individuals, and the covers of alien shrubs and lianas in individual plots separately against the chosen predictors. We used a mixed model framework with ‘trail’ as a random grouping factor that could account for some variation in the data. We had both count (trees, lianas) and proportions data (shrubs, herbs), so we used generalized linear mixed models to assign correct error distributions to the response data using the *glmmadmb* function of the *glmmADMB* package^51^. Because plant abundance data is often zero-inflated and prone to overdispersion, we tested several different, closely-related, error structures for count and proportions data, and chose between the models using Akaike Information Criterion (AIC) estimates. For count data we compared models with “Poisson”, “nbinom” and “nbinom1” errors, with zero inflation specified. For the proportions data we chose between “binomial” and “betabinomial”. We then compared nested models to remove predictors that were not explaining significant amounts of variation in the response variables, using AIC comparisons between larger and smaller models.

## Electronic supplementary material

Supplementary Info
