# Supplementary material for: Alien plant invasions of protected areas in Java, Indonesia
Source: Sci Rep. 2017 Aug 24;7:9334. doi: 10.1038/s41598-017-09768-z (PMC5570924; doi:10.1038/s41598-017-09768-z)
Supplement: Supplementary file 1 — Supplementary Info [file 41598_2017_9768_MOESM1_ESM.pdf]

Alien plant invasions of protected areas in Java, Indonesia

Michael Padmanaba, Kyle W. Tomlinson, Alice C. Hughes & Richard T. Corlett

Supplementary Table S1. Summary of the environmental parameter of the 403 plots in eight National Parks in Java.

Variables		UK	GP	MRP	MRB	BTS	MB	AP	BAL
Altitude (masl)	mean	46	2269	1851	2528	2231	113	43	65
	lowest	0	1402	1002	1859	861	21	0	0
	highest	463	2993	2658	3158	3100	279	100	267
Slope (º)	mean	3.3	18.2	17.1	15.8	8.4	2.4	3.1	3.4
	min	0.0	0.0	2.0	0.0	0.0	0.0	1.0	0.0
	max	16.0	57.0	42.0	34.0	47.0	7.0	11.0	23.0
Canopy cover (%)	mean	80.46	74.36	43.40	30.86	43.80	65.67	73.41	18.16
	lowest	7.30	0.00	0.00	0.00	0.00	0.00	23.20	0.00
	highest	97.80	97.90	90.00	92.50	100.00	91.20	84.00	95.30
Shrub cover (%)	mean	39.09	57.03	83.33	81.58	77.93	66.07	45.73	23.91
	lowest	0.00	0.00	0.00	25.00	0.00	25.00	25.00	0.00
	highest	100.00	100.00	100.00	100.00	100.00	100.00	100.00	100.00
Native shrub cover (%)	mean	38.70	44.10	64.90	60.10	51.80	53.40	43.50	19.10
	lowest	0.00	0.00	0.00	0.00	0.00	22.50	19.00	0.00
	highest	100.00	99.50	100.00	100.00	100.00	100.00	100.00	100.00
Sand (%)	mean	51.20	49.45	74.13	72.93	76.38	52.77	41.07	56.50
	lowest	47.00	35.10	69.02	65.08	62.07	24.01	16.03	19.01
	highest	53.00	65.82	79.95	79.94	88.97	78.99	56.00	62.00
Silt (%)	mean	30.33	15.10	19.45	20.97	11.66	27.52	22.46	15.89
	lowest	24.00	7.03	13.05	13.06	1.02	11.01	13.00	14.00
	highest	31.00	24.95	22.98	28.91	25.98	48.00	42.98	22.00
Clay (%)	mean	18.47	35.45	6.43	6.09	11.97	19.70	36.47	27.61
	lowest	16.00	23.12	4.03	4.00	7.01	10.00	31.00	22.00
	highest	29.00	46.99	8.00	7.99	17.94	28.00	48.97	58.99
pH	mean	5.73	5.11	5.74	5.81	5.98	6.49	7.55	8.55
	lowest	5.10	4.20	5.60	5.60	4.90	6.00	6.50	6.60
	highest	6.10	5.50	6.00	6.10	7.49	6.70	8.00	9.30
Total N (%)	mean	0.21	0.76	0.37	0.43	0.12	0.14	0.43	0.12
	lowest	0.17	0.54	0.19	0.19	0.02	0.08	0.07	0.09
	highest	0.25	1.11	0.51	0.69	0.20	0.19	0.67	0.19
Organic C (%)	mean	2.61	18.06	4.75	5.20	1.50	2.29	10.19	1.62
	lowest	2.35	1.02	2.47	2.47	0.16	1.14	1.20	1.01
	highest	3.39	29.17	6.48	7.48	2.63	3.70	16.74	3.32
Available P (ppm)	mean	17.03	47.98	10.92	11.52	16.96	21.53	30.84	17.09
	lowest	10.00	8.93	9.02	7.11	5.01	10.00	12.00	12.00
	highest	21.00	94.09	12.00	23.84	19.00	32.00	54.99	33.00
Available K (ppm)	mean	356.40	285.60	65.53	81.70	59.11	183.98	544.30	2015.20
	lowest	241.00	161.60	15.38	15.29	30.03	75.03	333.90	370.30
	highest	443.00	514.00	110.87	174.90	120.47	235.98	749.80	2680.00
Trail width (m)	mean	1.00	1.00	1.00	1.00	0.50	1.00	0.50	1.00
	minimum	0.92	1.86	0.88	1.76	3.86	1.86	2.11	4.34
	maximum	1.00	2.00	3.00	1.00	20.00	2.00	4.00	10.00

National Park acronyms are: UK=Ujung Kulon; GP=Gunung Gede Pangrango; MRP=Gunung Merapi; MRB=Gunung Merbabu; BTS=Bromo Tengger Semeru; MB=Meru Betiri; AP=Alas Purwo; BAL=Baluran.

Supplementary Table S2. Invasive alien plants species recorded in eight National Parks in Java ('+' indicates species found only outside the sample plots).

Species	Family	N of parks	N of plots	UK	GP	MRP	MRB	BTS	MB	AP	BAL
Acacia decurrens	Fabaceae	3	51			1	1	1			
Acacia nilotica	Fabaceae	1	21								1
Ageratina riparia	Asteraceae	4	120		1	1	1	1			
Ageratum conyzoides	Asteraceae	4	12			1	1	+	1		1
Artemisia vulgaris	Asteraceae	1	4					1			
Austroeupatorium inulaefolium	Asteraceae	4	48		1	1	1	1			
Ayapana triplinervis	Asteraceae	1	7			1					
Bartlettina sordida	Asteraceae	1	3		1						
Bidens pilosa	Asteraceae	3	17				1	1			1
Brugmansia candida	Solanaceae	1	5		1						
Brugmansia suaveolens	Solanaceae	1	9		+			1			
Calliandra houstoniana var. calothyrsus	Fabaceae	2	6			1		1			
Calopogonium mucunoides	Fabaceae	2	3						1		1
Carex lyngbyei	Cyperaceae	1	2		1						
Centrosema pubescens	Fabaceae	3	8					1	1		1
Cestrum aurantiacum	Solanaceae	2	17		1			1			
Chromolaena odorata	Asteraceae	7	109	1	+	1	1	1	1	1	1
Cleome aculeata	Cleomaceae	1	4								1
Cleome rutidosperma	Cleomaceae	1	4						1		
Clidemia hirta	Melastomataceae	6	7	1	1	1	1	1	1		
Crassocephalum crepidioides	Asteraceae	3	8		1	1	1				
Croton hirtus	Euphorbiaceae	2	7						1		1
Cuphea ignea	Lythraceae	1	1		1						
Elephantopus scaber	Asteraceae	2	3	+					1	+	1
Eleutheranthera ruderalis	Asteraceae	3	21				1		1		1
Erechtites hieraciifolius	Asteraceae	1	1				1				
Erigeron sumatrensis	Asteraceae	2	4			1	1				
Euphorbia heterophylla	Euphorbiaceae	2	9						1		1
Euphorbia hirta	Euphorbiaceae	2	4	+					1		1
Foeniculum vulgare	Apiaceae	1	3					1			
Fuchsia magellanica	Onagraceae	1	10					1			
Hyptis capitata	Lamiaceae	4	12	1					1	1	1
Hyptis suaveolens	Lamiaceae	1	8								1
Jatropha curcas	Euphorbiaceae	1	2								1
Jatropha gossypiifolia	Euphorbiaceae	1	5								1
Lantana camara	Verbenaceae	7	25	1	+	1	1	1	1	1	1
Leucaena leucocephala	Fabaceae	2	11					+	1		1
Maranta lietzei	Marantaceae	1	1	1	+						
Mikania micrantha	Asteraceae	2	12						1	1	
Mimosa diplotricha	Fabaceae	2	3						1		1
Mimosa pudica	Fabaceae	2	6						1	+	1
Oxalis barrelieri	Oxalidaceae	1	3	+					1		
Oxalis debilis var. corymbosa	Oxalidaceae	1	3				1				
Paspalum conjugatum	Poaceae	1	1							1	

Passiflora foetida	Passifloraceae	3	29	1					1	+	1
Passiflora ligularis	Passifloraceae	1	4		1						
Pennisetum purpureum	Poaceae	3	6			1	1	1			
Physalis pruinosa	Solanaceae	1	3					1			
Phytolacca icosandra	Phytolaccaceae	1	12					1			
Plantago major	Plantaginaceae	2	4		1			1			
Rhynchospora colorata	Cyperaceae	2	7			1			1		
Ruellia tuberosa	Acanthaceae	1	2								1
Senna obtusifolia	Fabaceae	1	1						1		
Sida rhombifolia	Malvaceae	3	5						1	1	1
Solanum americanum	Solanaceae	2	9		1			1			
Solanum giganteum	Solanaceae	1	1		1						
Solanum torvum	Solanaceae	2	8					1	1		
Sonchus asper	Asteraceae	1	5					1			
Spermacoce alata	Rubiaceae	1	3						1		
Spermacoce laevis	Rubiaceae	1	3						1		
Spigelia anthelmia	Loganiaceae	1	1	1							
Stachytarpheta jamaicensis	Verbenaceae	3	9	1				+	1	1	
Synedrella nodiflora	Asteraceae	2	2		1					1	
Tithonia diversifolia	Asteraceae	1	3					1			
Tridax procumbens	Asteraceae	2	2						1		1
Verbena brasiliensis	Verbenaceae	1	15					1			
Zapoteca portoricensis	Fabaceae	1	5								1

Supplementary Table S3. Native distribution and date of first record in Java for the alien plant species recorded in this study.

Species	Life form	Native to	First record in Java
Acacia decurrens	Tree	Australia	1866
Acacia nilotica	Tree	Africa and West Asia	1866
Ageratina riparia	Herb	Mexico, West Indies	1963
Ageratum conyzoides	Herb	Tropical America	1844
Artemisia vulgaris	Herb	Europe, North America	1844
Austroeupatorium inulaefolium	Shrub	South America	1900s
Ayapana triplinervis	Herb	Brazil	1844
Bartlettina sordida	Shrub	Mexico	1899
Bidens pilosa	Herb	Tropical America	1844
Brugmansia candida	Shrub	Peru	1963
Brugmansia suaveolens	Shrub	Brazil	1866
Calliandra houstoniana var. calothyrsus	Tree	Tropical America	1963
Calopogonium mucunoides	Liana	Central and South America	1920s
Carex lyngbyei	Herb	North America	
Centrosema pubescens	Herb	South America	1922
Cestrum aurantiacum	Shrub	Central America	1866
Chromolaena odorata	Shrub	Central and South America	1940s
Cleome aculeata	Herb	Tropical America	1866
Cleome rutidosperma	Herb	Tropical Africa	1946
Clidemia hirta	Shrub	Tropical America	late 19th century
Crassocephalum crepidioides	Herb	Tropical Africa	1930s
Croton hirtus	Herb	Tropical America	before 1900
Cuphea ignea	Herb	Mexico, West Indies	1866
Elephantopus scaber	Herb	West Indies and Tropical America	1844
Eleutheranthera ruderalis	Herb	Tropical America	1888
Erechtites hieraciifolius	Herb	Tropical America	1863
Erigeron sumatrensis	Herb	Tropical America	before 1860
Euphorbia heterophylla	Herb	Tropical America	1963
Euphorbia hirta	Herb	Southern USA and tropical America	1963
Foeniculum vulgare	Herb	South Europe	1844
Fuchsia magellanica	Shrub	Temperate South America	1866
Hyptis capitata	Herb	Tropical America	1880
Hyptis suaveolens	Herb	Tropical America	1844
Jatropha curcas	Shrub	Tropical America	1844
Jatropha gossypiifolia	Shrub	Tropical America	1866
Lantana camara	Shrub	Central and South America	1866
Leucaena leucocephala	Tree	Tropical America	1844
Maranta lietzei	Herb	Tropical America	1963
Mikania micrantha	Liana	Central and South America	1949
Mimosa diplotricha	Shrub	Tropical America	early 20th century
Mimosa pudica	Shrub	Tropical America	1844
Oxalis barrelieri	Herb	South America	1888
Oxalis debilis var. corymbosa	Herb	South America, West Indies	1844
Paspalum conjugatum	Herb	Tropical America	1963

Passiflora foetida	Liana	Tropical America	1963
Passiflora ligularis	Liana	Tropical America	1963
Pennisetum purpureum	Herb	Tropical Africa	1963
Physalis pruinosa	Herb	Central and South America	1963
Phytolacca icosandra	Shrub	Tropical America	1866
Plantago major	Herb	Europe, Central Asia	1866
Rhynchospora colorata	Herb	North and Central America, West Indies	1963
Ruellia tuberosa	Herb	Central and South America	before 1901
Senna obtusifolia	Shrub	Tropical America	1963
Sida rhombifolia	Shrub	North America	1844
Solanum americanum	Herb	Southern USA and tropical America	1844
Solanum giganteum	Shrub	East Africa	1866
Solanum torvum	Shrub	Central and South America	1963
Sonchus asper	Herb	Europe, North Africa	1844
Spermacoce alata	Herb	Tropical America	1963
Spermacoce laevis	Herb	Tropical America	1963
Spigelia anthelmia	Herb	Tropical America	1866
Stachytarpheta jamaicensis	Shrub	Tropical America	1866
Synedrella nodiflora	Herb	Tropical America	1888
Tithonia diversifolia	Herb	Mexico, Central America	before 1900
Tridax procumbens	Herb	Tropical America	1866
Verbena brasiliensis	Herb	Tropical America	1963
Zapoteca portoricensis	Shrub	Central and South America, West Indies	1963

Note: 1844 = Listed in Bogor Botanic Garden catalogue (Hasskarl, 1844); 1866 = Listed in Bogor Botanic Garden catalogue (Teijsmann and Binnendijk, 1866); 1963 = Listed in Backer and van Den Brink (1963).

Supplementary Table S4. Trails surveyed in the eight National Parks in Java.

National Park	Land area (km^2)	Trail	Length (km)	Total Plots	Vegetation types
UK	786.2	UK1	33	33	forest
		UK2	18	18	forest
		UK3	4	4	forest
GP	219.75	GP1	10	19	forest, open area at the top
		GP2	2	4	forest, open area at the top
		GP3	5	9	forest, open area at the top
MRP	64.1	MRP1	3.5	14	forest
		MRP2	2	8	open forest
		MRP3	5	21	forest, open area at the top
		MRP4	1.5	5	forest
MRB	57.25	MRB1	4.5	18	forest, open area at the top
		MRB2	5	20	forest, open area at the top
BTS	502.76	BTS1	14	30	forest and open area
		BTS2	4.5	10	forest
		BTS3	4	7	forest
		BTS4	4	8	forest
		BTS5	7	14	open area
		BTS6	10	20	forest
		BTS7	2.5	5	forest
MB	553.97	MB1	8	7	forest
		MB2	9	7	forest
AP	430.2	AP1	12	10	forest
		AP2	4	5	forest
		AP3	2	2	savanna
		AP4	16	16	forest
		AP5	2	2	forest
		AP6	2	2	open forest
		AP7	3.5	4	forest
BAL	239.37	BAL1	21	23	savanna
		BAL2	36.5	39	savanna
		BAL3	10	10	forest
		BAL4	7.5	9	forest

National Park acronyms are: UK=Ujung Kulon; GP=Gunung Gede Pangrango; MRP=Gunung Merapi; MRB=Gunung Merbabu; BTS=Bromo Tengger Semeru; MB=Meru Betiri; AP=Alas Purwo; BAL=Baluran.

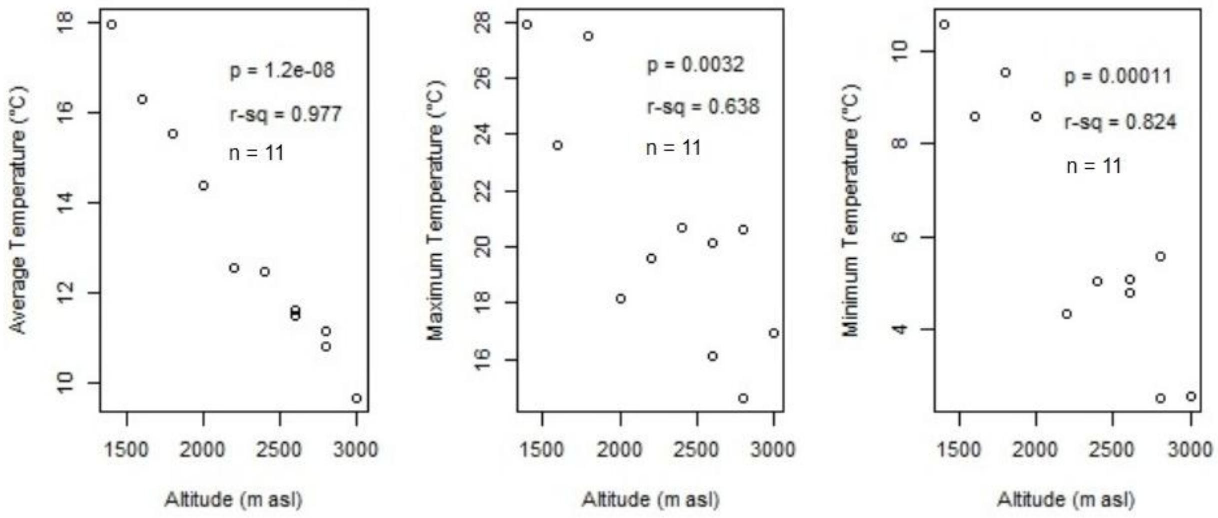

Supplementary Figure S1. Mean, maximum, and minimum temperatures measured over one year at different altitudes in Gunung Gede Pangrango National Park.

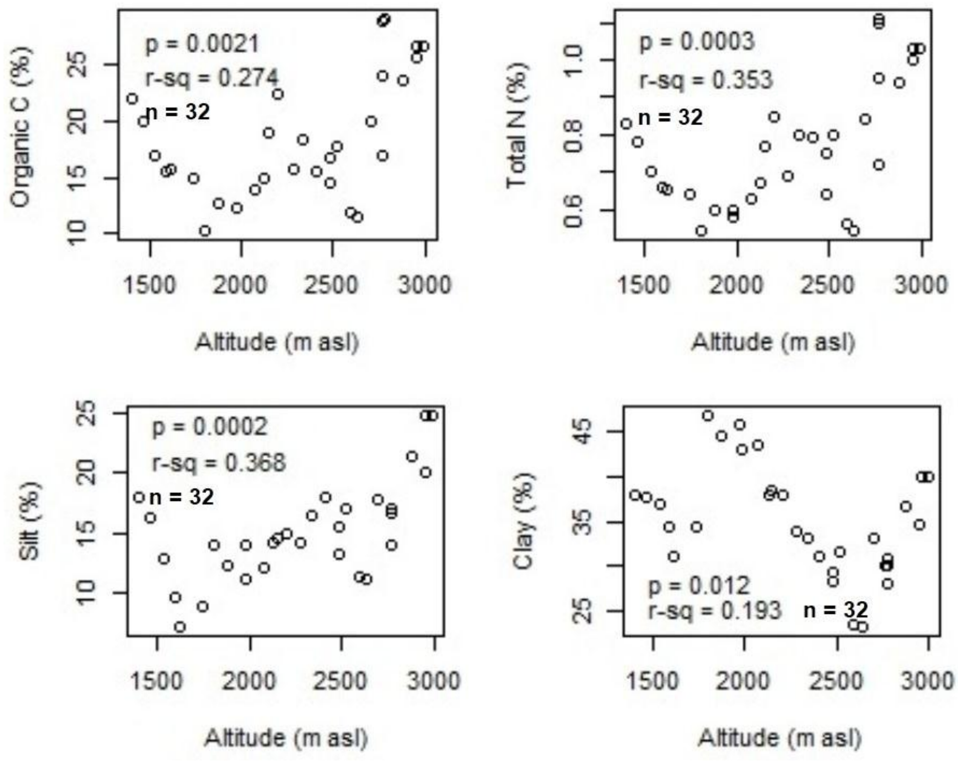

Supplementary Figure S2. Organic carbon, nitrogen, silt, and clay percentages at different altitudes in Gunung Gede Pangrango National Park.
